# The Uric Acid-to-High-Density Lipoprotein Cholesterol Ratio: A New Biomarker for Predicting Arrhythmia Recurrence After Atrial Fibrillation Ablation

**DOI:** 10.3390/jcm13247854

**Published:** 2024-12-23

**Authors:** Emir Dervis, Eyup Ozkan, Idris Yakut, Hasan Can Konte, Aykun Hakgor, Omer Alyan, Taylan Akgun, Dursun Aras

**Affiliations:** 1 Department of Cardiology, Medipol University, Istanbul 34810, Türkiye; idrislive@windowslive.com (I.Y.); hcankonte@gmail.com (H.C.K.); aykunhakgor@gmail.com (A.H.); droalyan@yahoo.com (O.A.); drdaras@gmail.com (D.A.); 2 Department of Cardiology, Basaksehir Cam and Sakura City Hospital, Istanbul 34480, Türkiye; dreyupozkan@gmail.com (E.O.); taylanakgun@gmail.com (T.A.)

**Keywords:** uric acid, high-density lipoprotein cholesterol, atrial fibrillation, ablation techniques, recurrence

## Abstract

**Background:** We aimed to assess the uric acid-to-high-density lipoprotein cholesterol (HDL-C) ratio (UHR) and several other parameters with respect to their performance in detecting recurrence among patients with atrial fibrillation (AF) who underwent ablation. **Methods:** This retrospective cohort study analyzed data from patients who underwent radiofrequency or cryoablation for paroxysmal, persistent, or long persistent AF between September 2021 and September 2023. After ablation, patients were monitored for 24 h, with an ECG Holter used for symptomatic cases. Follow-up visits occurred at 1, 3, and 12 months. Collected data included demographics, comorbidities, echocardiographic measurements, clinical data, ablation type, medication use, and a comprehensive set of laboratory findings. **Results:** The study included 163 patients, with AF recurrence in 39 (23.93%) patients. Mean age was 57.49 ± 11.22 years, and 59.51% of participants were male. There was no significant difference between recurrent and non-recurrent groups in terms of age or sex distribution. Univariate analysis showed that recurrent patients had significantly larger left atrium diameter, higher percentages of persistent/long AF, and elevated levels of CRP, uric acid, UHR, and uric acid-to-creatinine ratio (UCR). Logistic regression analysis revealed that high left atrium diameter, long persistent AF presence, high CRP and uric acid levels, and high UCR and UHR values greater than 15.1 were independent predictors of AF recurrence. A UHR value of >15.1 was found to predict recurrence with 61.54% sensitivity and 70.97% specificity. **Conclusions:** Despite low sensitivity, UHR appears to be an independent biomarker that can predict AF recurrence. Including UHR in future risk assessment tools may be beneficial to enhance their accuracy.

## 1. Introduction

Atrial fibrillation (AF) is the most common type of arrhythmia encountered in clinical practice and causes significant morbidity and mortality [1,2,3]. The Global Burden of Disease 2019 study described an increasing trend in the global prevalence of AF [4], which is projected to continue over the next thirty years [5].

Atrial fibrillation is mechanistically complex and available treatments targeting the underlying activity are often inadequate. For instance, approximately 70% of patients experience recurrence after cardioversion [6]. This proportion can be somewhat reduced with the use of antiarrhythmic drugs; however, antiarrhythmic drug therapy is of limited efficacy as it targets only the electrical circuitry and has several side effects and proarrhythmic risks [7].

Atrial fibrillation is categorized into paroxysmal, persistent, and long-standing persistent types, each with varying recurrence risks and treatment strategies [2,3]. Current guidelines recommend catheter ablation as an effective approach for rhythm control, particularly in patients with symptomatic, drug-refractory AF [2,3]. Catheter ablation provides better quality of life and better left ventricular function in patients with heart failure compared to antiarrhythmic drug therapy, and these benefits are significantly associated with whether the patient remains free of AF [8]. However, the recurrence rate of AF after catheter ablation is estimated to be between 20% and 45% within the first year, depending on patient-specific factors and AF type [9], highlighting the growing research interest in identifying patients at risk of developing recurrence after ablation. Several risk factors and predictive models have been reported to predict recurrent AF after ablation, including age, hypertension, diabetes, heart failure, subclinical hyperthyroidism, obesity, sleep apnea syndrome, type of AF (chronic or paroxysmal), and certain echocardiographic parameters [10,11,12,13,14]. Since elevated inflammation plays a crucial role in both the initiation and progression of AF [15,16,17], there is an ongoing interest in investigating different inflammatory markers as predictors of recurrence.

Increased uric acid production is believed to induce oxidative stress and chronic low-grade inflammation and elevated uric acid has been associated with vascular endothelial dysfunction, increased formation of advanced glycation end products, and activation of the renin-angiotensin system [18,19,20,21]. High-density lipoprotein cholesterol (HDL-C) removes excess cholesterol from peripheral tissues and transports it to the liver [16]. Additionally, HDL-C plays a role in suppressing oxidation reactions in the blood and protecting vascular endothelial cells [22,23,24]. The uric acid-to-HDL-C ratio (UHR) has gained renown as a potential biomarker associated with metabolic and cardiovascular diseases. Previous studies have shown that the UHR is associated with hypertension control, [25] hepatic steatosis [26], thyroiditis [27], cardiovascular mortality [28], metabolic syndrome [29], and arrhythmias [30]. Although serum uric acid levels and lipid levels have been individually investigated in relation to AF occurrence and recurrence in previous studies [19,20,22,31], to our knowledge, the relationship between UHR and AF recurrence after ablation has not been explored.

We hypothesized that UHR could predict AF recurrence after ablation and aimed to investigate the effects of UHR, a valuable biomarker indicative of chronic inflammation and metabolic status, and several other clinical and laboratory parameters on the prediction of recurrence in patients undergoing AF ablation.

## 2. Materials and Methods

### 2.1. Ethical Considerations

This retrospective study was started after receiving approval from the Non-Interventional Clinical Research Ethics Committee of Istanbul Medipol University (Decision date: 13 June 2024, decision no: 579). It was performed in line with the principles of the Declaration of Helsinki.

### 2.2. Setting and Population

Patient recruitment and all study-related analyses were conducted at Basaksehir Cam and Sakura City Hospital and Istanbul Medipol University Cardiology Department, Istanbul, Turkey. All patients who underwent radiofrequency ablation or cryoablation due to paroxysmal, persistent, or long-persistent AF, between September 2021 and September 2023, were evaluated for inclusion. Patients with missing data, those who did not continue outpatient follow-up, those with an ejection fraction below 35%, recipients of repeat ablation, those without 1-year follow-up, patients with known inflammatory diseases that could impact analysis results, those using xanthine oxidase inhibitor therapy and those with a known history of immunosuppressive drug use before ablation were excluded from the study.

### 2.3. Data Collection and Tools

Patient data including age, sex, height, weight, smoking status (non-smoker, ex-smoker, active smoker), comorbidity status, echocardiographic measurements (ejection fraction and left atrium diameter), AF type (paroxysmal, persistent or long persistent), type of ablation procedure performed (radiofrequency ablation or cryoablation), anticoagulant and antiarrhythmic use, and laboratory findings [C-reactive protein (CRP), albumin, creatinine, uric acid, triglyceride, total cholesterol, HDL-C, low-density lipoprotein cholesterol (LDL-C) levels, and hemoglobin, platelet, neutrophil, lymphocyte, and monocyte counts] were obtained through a retrospective review of hospital records.

Body mass index (BMI, kg/m^2^) was calculated by dividing weight (kg) by the square of height (m^2^). CHA_2_DS_2_-VASc scores were also calculated.

All laboratory findings were obtained from routine blood tests performed prior to ablation. Analyses were conducted in our hospital’s certified biochemistry laboratories using regularly calibrated routine measurement devices in accordance with international standards. The uric acid level was divided by the HDL-C level to obtain UHR (%). Based on the median value, patients were grouped into low and high UHR. To assess the impact of serum uric acid levels normalized for kidney function, serum uric acid levels were divided by creatinine levels to calculate the serum uric acid/creatinine ratio (UCR).

### 2.4. Diagnosis and Definitions

All patients underwent detailed medical history taking and comprehensive physical examination prior to the procedure. Diagnostic tests such as transthoracic echocardiography and electrocardiography were used to determine the type and severity of AF.

Paroxysmal AF was defined as AF that resolves spontaneously within 48 h or with medical or electrical cardioversion within seven days. Persistent AF is defined as AF episodes lasting longer than seven days but less than one year, requiring medical intervention for termination. Long persistent AF was defined as AF that persisted continuously for more than one year despite attempts at rhythm control [32].

### 2.5. Ablation Procedures and Follow-Up

Patients were stabilized with antiarrhythmic drug therapy before the ablation procedure. On the day of the procedure, local anesthesia and sedation were administered, and a catheter was inserted via the femoral vein.

For patients undergoing radiofrequency ablation, the Carto 3-dimensional electroanatomic mapping system was utilized to accurately delineate the anatomy of the pulmonary veins. Subsequently, an irrigated-tip radiofrequency ablation catheter (ThermoCool SmartTouch, Biosense Webster, Diamond Bar, CA, USA) was employed to deliver radiofrequency energy, thereby achieving pulmonary vein isolation. During this procedure, the catheter tip applied high-frequency electrical energy to heat the targeted tissues, effectively disabling the abnormal electrical pathways.

For patients undergoing cryoablation, a cryoballoon ablation catheter (Arctic Front Advance, AFA-Pro, Medtronic, Minneapolis, MN, USA) was used to map and isolate the pulmonary veins. In this process, the catheter tip was cooled (−50 °C to −70 °C) to specifically target and permanently disable the abnormal electrical pathways responsible for AF.

Following the procedure, all patients were monitored under cardiac observation for a minimum of 24 h to assess for potential complications. In cases where patients reported symptoms after ablation, a 24 h ECG Holter monitor was employed to detect any AF episodes. Patients were subsequently followed by regular clinical assessments and ECG rhythm monitoring throughout the recovery period. Scheduled cardiology outpatient clinic visits were conducted at 1-, 3-, and 12-months post-ablation. Additionally, patients were instructed to return to the outpatient clinic if they experienced any symptoms. Monitoring for AF recurrence was carried out, with the first-month post-ablation considered a blanking period, during which any AF or atrial flutter occurrences were not classified as recurrences. Patients who developed AF or atrial flutter within the first 3 months were classified as having early recurrence, while those who experienced it between 3 months and 1 year were classified as having late recurrence [33].

### 2.6. Statistics

Data were collected into an SPSS v25.0 database, and analyses were performed with the same software based on the classical significance threshold of 0.05 alpha error (*p*-value) (IBM Corporation, Armonk, NY, USA). Histograms and Q-Q plots were utilized to assess the conformity of continuous variables to a normal distribution. Descriptive statistics were reported as mean ± standard deviation for normally distributed continuous variables, median (25th percentile–75th percentile; IQR) for non-normally distributed continuous variables, and frequency (percentage) for categorical variables. Comparative analysis of continuous variables between groups was conducted using either Student’s *t*-test or the Mann–Whitney U test, depending on the normality of the distribution. Categorical variables were compared between groups using the chi-square test, Fisher’s exact test, or its Freeman–Halton extension.

The predictive performance of the UHR and UCR for recurrence was evaluated through receiver operating characteristic (ROC) curve analysis. The optimal cut-off point was identified using the Youden index. Sensitivity, specificity, accuracy, positive predictive value, and negative predictive value were calculated based on the determined cut-off point. To identify significant factors independently associated with recurrence, multivariable logistic regression analysis was conducted. Three separate multivariable logistic regression models were constructed for uric acid, uric acid-to-HDL-C ratio, and uric acid-to-creatinine ratio to avoid multicollinearity problems. Variables that were statistically significant in univariate analysis were included in the multivariable logistic regression model.

## 3. Results

A total of 163 patients were included in the study. AF recurrence occurred in 39 (23.93%) patients and the median recurrence time was 3 (IQR: 2–4, range: 1–12) months. The mean age of all participants was 57.49 ± 11.22 years and 97 (59.51%) were male. There was no significant difference between the recurrent and non-recurrent groups in terms of age (*p* = 0.502) and sex (*p* = 0.311). The recurrent group had significantly higher values for mean left atrium diameter (*p* < 0.001), percentage of patients with persistent and long AF (*p* = 0.001), mean CRP level (*p* = 0.017), mean uric acid (*p* = 0.002), UHR (*p* = 0.002) and UCR (*p* = 0.009) levels (Table 1).

UHR values were able to significantly discriminate between patients with and without AF recurrence. ROC analysis results revealed an AUC of 0.665 (95% CI: 0.562–0.768, *p* = 0.002), and the >15.1 cut-off had 61.54% sensitivity and 70.97% specificity to identify patients with recurrence. Also, UCR values were able to significantly discriminate between patients with and without AF recurrence. ROC analysis results revealed an AUC of 0.671 (95% CI: 0.572–0.770, *p* = 0.001), and the >6.45 cut-off had 71.79% sensitivity and 61.29% specificity to identify patients with recurrence (Table 2).

According to the median value of UHR, we divided the patients into two groups: “low UHR, ≤13.57 (n = 82)” and “high UHR, >13.57 (n = 81)”. The High UHR group had significantly higher mean weight (*p* = 0.005), BMI (*p* = 0.001), uric acid (*p* < 0.001), and UCR levels (*p* < 0.001), median triglyceride levels (*p* = 0.004), and significantly lower mean HDL-C (*p* < 0.001), as well as a higher recurrence rate (*p* = 0.009) compared to the low UHR group (Table 3).

According to the logistic regression analysis results; in model 1, which included only uric acid among uric acid, UHR, and UCR, the following were independently associated with recurrence: high left atrium diameter (OR: 1.227, 95% CI: 1.074–1.402, *p* = 0.003), long persistent AF (OR: 2.633, 95% CI: 1.061–6.533, *p* = 0.037), and uric acid level (OR: 1.464, 95% CI: 1.095–1.958, *p* = 0.010). The other variable included in model 1, namely CRP (*p* = 0.104), was found to be non-significant. In model 2, which included only UHR among uric acid, UHR, and UCR, the following were independently associated with recurrence: high left atrium diameter (OR: 1.212, 95% CI: 1.063–1.381, *p* = 0.004), long persistent AF (OR: 2.660, 95% CI: 1.050–6.740, *p* = 0.039), and high UHR (>15.1) level (OR: 3.824, 95% CI: 1.682–8.693, *p* = 0.001). The other variable included in model 2, namely CRP (*p* = 0.081), was found to be non-significant. In model 3, which included only UCR among uric acid, UHR, and UCR, the following were independently associated with recurrence: high left atrium diameter (OR: 1.189, 95% CI: 1.039–1.360, *p* = 0.012), long persistent AF (OR: 2.722, 95% CI: 1.065–6.959, *p* = 0.037), CRP (OR: 1.018, 95% CI: 1.003–1.032, *p* = 0.015), and high UCR (>6.45) level (OR: 4.340, 95% CI: 1.768–10.652, *p* = 0.001) (Table 4).

## 4. Discussion

Catheter ablation of AF has become a well-established and widely used therapy [34]. However, arrhythmia recurrences after ablation are common, and many patients face the risk of recurrent AF and need repeat ablation [18,35,36]. Therefore, identifying patients with a high risk of recurrent AF after ablation is crucial. This study investigated the predictability of recurrence after AF ablation, focusing on UHR and other risk factors. Our data shows that high left atrium diameter, long persistent AF, CRP, uric acid, high UHR high UCR were independently associated with AF recurrence. Patients with high UHR and high UCR had higher AF recurrence rates. UHR was able to significantly predict AF recurrence after AF ablation. Despite having a lower-than-desired level of sensitivity, the reliable specificity and the high negative predictive value of UHR indicate that it may have utility in determining the likelihood of AF recurrence after ablation.

Although the exact mechanism of AF remains unclear [37,38], there is evidence that inflammatory responses and oxidative stress play significant roles in the pathogenesis [39]. Uric acid is the final metabolite of purine and is catalyzed by xanthine oxidase, which is a major source of reactive oxygen species (ROS) [18]. Serum uric acid level, as a molecular marker of inflammation and oxidative stress, has been found to be associated with the occurrence and progression of AF [18,19,20,21]. However, studies on serum uric acid levels in patients with and without AF have yielded contradictory results [18]. Recent meta-analyses have reported that hyperuricemia increases the risk of AF [19,20], which are results that have been obtained from various studies. For instance, Wang et al. demonstrated that high serum uric acid levels were associated with the incidence of AF and its duration, while individuals with persistent AF had significantly higher levels [40]. Furthermore, uric acid-lowering therapies have been shown to inhibit the progression of AF by preventing cardiac remodeling [41].

The analysis of relationships between pre-ablation uric acid levels and post-ablation AF recurrence has yielded contradictory results. The results of a meta-analysis showed that the serum uric acid level in the patients with AF recurrence after ablation group was significantly higher than in the non-recurrence group [42]. A study conducted in Turkey found that high pre-ablation serum uric acid levels were an independent predictor of AF recurrence after cryoballoon-based catheter ablation [16]. On the other hand, two separate meta-analyses [40,43] reported no link between serum uric acid level and AF recurrence after ablation [40]. Similarly to the output of these meta-analyses, our univariate analysis showed higher uric acid levels in the recurrence group, but these associations disappeared with multivariable analysis. These differing results have been suggested to be due to the heterogeneity of study populations among the studies [40,42]. The possible relationship between uric acid levels and the occurrence, persistence, and recurrence of AF may arise from the following potential mechanisms. (i) Uric acid production generates ROS, increasing oxidative stress, which can lead to electrical and structural changes in atrial tissue, contributing to AF pathophysiology (ii). With a similar mechanism, the inflammatory response triggered by high uric acid can lead to fibrosis and scar formation in atrial tissue, altering electrical pathways. (iii) Uric acid-triggered endothelial dysfunction can increase vascular reactivity and inflammation which lead to tissue abnormalities that have a direct impact, while (iv) the resultant structural remodeling due to these changes may also facilitate both the onset and recurrence of atrial fibrillation. (v) Finally, oxidative stress and inflammation can affect the function of ion channels in atrial myocytes, which may incite abnormal electrical activity in atrial cells, triggering AF [18,19,20,42]. These mechanisms can help us understand the impact of uric acid levels on the occurrence, frequency, and especially post-ablation recurrence of AF, as well as the role of uric acid-lowering therapies in the occurrence and recurrence of AF.

The relationship between dyslipidemia and AF is controversial. Numerous studies have shown that dyslipidemia is inversely associated with AF incidence [22,31], suggesting that lower cholesterol levels are associated with a higher risk of AF. However, contrary to previous studies, a combined analysis from the MESA-FHS cohorts showed that HDL-C and triglycerides were associated with AF risk, but LDL-C or total cholesterol were not [44]. Shang et al. demonstrated that lower total cholesterol and LDL-C levels were associated with post-ablation AF recurrence in women, but HDL-C and triglyceride levels were not associated with AF recurrence in either sex [23]. In contrast, a recent meta-analysis reported that the group with AF recurrence after radiofrequency catheter ablation had higher LDL-C levels [19]. In our study, AF recurrence was unassociated with triglyceride, total cholesterol, HDL-C, and LDL-C levels. Nonetheless, there may be several mechanisms that could facilitate links. Dyslipidemia can lead to increased oxidative stress and the impact on bioactive lipids could alter cellular pathways [24,45]. Dyslipidemia in relation to obesity can also cause structural changes in atrial tissue (e.g., atrial enlargement) [46], which can underlie the occurrence of AF. Alterations in structural lipid components also have the risk of restricting tissue healing and physiological remodeling mechanisms after ablation, disrupting the structural integrity of the atrial tissue [47,48]. Considering the possible mechanisms between AF and the levels of uric acid and lipids, it is feasible to suggest that UHR has the potential to provide a broader insight.

The pathophysiology of AF, which is not fully understood, suggests that it may be related to various mechanisms. In this context, various studies have investigated the relationship between AF and/or AF recurrence after ablation and the ratios of biomarkers representing different mechanisms. As a result of these studies, serum uric acid-to-creatinine ratio [49], uric acid-to-albumin ratio [15], monocyte-to-HDL-C ratio [16], CRP-to-albumin ratio [12], and neutrophil-to-lymphocyte ratio [17] have all been associated with the recurrence of AF after catheter ablation. The present study demonstrated that high UHR is an independent risk factor for AF recurrence. A high UHR (>15.1) predicted AF recurrence with 61.54% sensitivity and 70.97% specificity. Previous studies have suggested that UHR can be considered an important biomarker in the risk assessment and monitoring of some cardiovascular and metabolic diseases [25,26,27,28,29,30]. Our study shows that UHR is independently associated with AF recurrence despite the fact that neither uric acid nor HDL-C levels alone were individually associated with recurrence. This can be explained by the complex mechanisms involved in AF and the fact that UHR may facilitate a simplified analysis of the impacts exerted by uric acid and lipid alterations, which could be involved in the pathophysiology. A high UHR could indicate increased inflammation, elevated oxidative stress, increased risk of fibrosis in atrial tissue, increased endothelial dysfunction, and increased metabolic and cardiovascular risks. These suggest that patients with high UHR could require closer monitoring following ablation.

Alongside UHR, independent variables that could predict AF recurrence were identified as long persistent AF, increased left atrium diameter, CRP, uric acid, and high UCR consistent with established risk factors. Other studies have found age [12], obstructive sleep apnea [13], obesity [14], left ventricular filling pressure [10], AF type [50], pulmonary arterial hypertension [50], LDL-C [50], female sex [11], arterial hypertension [11], CHA_2_DS_2_-VASc score [11], and the presence of gout [11] to be associated with AF recurrence. In addition to many independent risk factors, various risk-scoring models have been developed to predict arrhythmia recurrence after AF ablation. Among the most frequently used are the APPLE score (age, type of AF, estimated glomerular filtration rate (eGFR), left atrial diameter, and left ventricular ejection fraction), ALARMEc score (type of AF, metabolic syndrome, eGFR, and normalized left atrial area), BASE-AF2 (type of AF, left atrial diameter, BMI, current smoking, AF history, and early recurrence), ATLAS (age, sex, type of AF, current smoking, and indexed left atrial volume), CAAP-AF (age, sex, type of AF, left atrial diameter, coronary artery disease, and number of antiarrhythmic drugs failed), HATCH (age, heart failure, hypertension, chronic obstructive pulmonary disease, and stroke/transient ischemic attack), MB-LATER (sex, type of AF, left atrial diameter, early recurrence, and bundle branch block), FER2CI score (sex, coupling interval of atrial premature contraction, and early recurrence) and SUCCESS score (APPLE + previous ablation) [18]. However, a systematic review of these scoring systems suggests that there remains a need for robust evaluation of risk factors and the development of better risk scores [18]. Zhang et al. demonstrated that elevated preoperative UCR is associated with the recurrence of AF after catheter ablation [49]. In a recent study, in patients discharged for acute heart failure serum uric acid-to-eGFR ratio was associated with poor cardiovascular outcomes, including arrhytmias [51]. The results of our study supported that UCR could predict AF recurrence after catheter ablation. It is evident that the risk factors identified for AF recurrence and the existing scoring systems are insufficient, indicating the need for new data. Identifying more reliable risk factors for AF recurrence after ablation would allow better patient selection with higher success rates and could provide insights into developing new preventive treatments. The UHR is one such candidate parameter that can improve risk-scoring systems to be developed in the future.

### Study Limitations

This study is the first report presenting data on the ability of UHR to predict AF recurrence after ablation. However, some limitations should be considered when interpreting the results. As a single-center study, its external validity is limited. It also possesses all the potential limitations of a retrospective study. One notable limitation is the inability to include new parameters such as physical activity, and alcohol consumption; however, the lack of these parameters would not have impacted the results obtained by UHR. Another limitation is that the study investigated recurrence within 12 months after ablation and did not explore late AF recurrence; however, it is clear that it would be ineffective to continue to assess the discriminatory capacity of a parameter that was calculated with data measured before ablation. In relation to this, all variables included in the study were measured before ablation, not during follow-ups. Therefore, there is a need for long-term longitudinal studies to investigate UHR over extended follow-up periods.

## 5. Conclusions

The present study identified high UHR, high UCR, high CRP, and uric acid levels, increased left atrium diameter, and long persistent AF as independent risk factors for AF recurrence after ablation. UHR was able to significantly predict AF recurrence, with low sensitivity but reliable specificity. UHR appears to be an important independent biomarker for predicting AF recurrence and could be valuable to include in future risk-scoring systems to increase their effectiveness. However, the findings need to be supported by more comprehensive studies that preferably examine UHR levels in a longitudinal manner.

## Figures and Tables

**Table 1:** Summary of patients’ characteristics, treatment protocols, and laboratory measurements with regard to recurrence.

		Recurrence		
	Total (n = 163)	No (n = 124)	Yes (n = 39)	*p*
Age, years	57.49 ± 11.22	57.77 ± 11.91	56.59 ± 8.71	0.502 ^†^
Sex				
Female	66 (40.49%)	47 (37.90%)	19 (48.72%)	0.311 ^#^
Male	97 (59.51%)	77 (62.10%)	20 (51.28%)	
Weight, kg	84.70 ± 13.77	85.10 ± 14.60	83.41 ± 10.74	0.504 ^†^
Height, cm	170.24 ± 9.72	170.57 ± 10.02	169.18 ± 8.74	0.437 ^†^
Body mass index, kg/m^2^	29.23 ± 4.19	29.24 ± 4.35	29.19 ± 3.66	0.947 ^†^
Smoking status				
Non-smoker	88 (53.99%)	62 (50.00%)	26 (66.67%)	0.185 ^#^
Ex-smoker	43 (26.38%)	36 (29.03%)	7 (17.95%)	
Active smoker	32 (19.63%)	26 (20.97%)	6 (15.38%)	
Diabetes mellitus	38 (23.31%)	27 (21.77%)	11 (28.21%)	0.541 ^#^
Hypertension	87 (53.37%)	65 (52.42%)	22 (56.41%)	0.801 ^#^
Cerebrovascular disease	11 (6.75%)	7 (5.65%)	4 (10.26%)	0.296 ^§^
Heart failure	12 (7.36%)	10 (8.06%)	2 (5.13%)	0.733 ^§^
Coronary artery disease	36 (22.09%)	30 (24.19%)	6 (15.38%)	0.350 ^#^
CHA_2_DS_2_-VASc score	2 (1–3)	2 (1–3)	2 (1–3)	0.806 ^‡^
Ejection fraction	60 (55–62)	60 (55–62)	61 (52–63)	0.576 ^‡^
Left atrium diameter	42.71 ± 4.26	41.97 ± 4.52	45.08 ± 1.90	<0.001 ^†^
AF type				
Paroxysmal	75 (46.01%)	67 (54.03%)	8 (20.51%) *	0.001 ^#^
Persistent	56 (34.36%)	38 (30.65%)	18 (46.15%)	
Long persistent	32 (19.63%)	19 (15.32%)	13 (33.33%) *	
Intervention				
Radiofrequency ablation	71 (43.56%)	51 (41.13%)	20 (51.28%)	0.352 ^#^
Cryoablation	92 (56.44%)	73 (58.87%)	19 (48.72%)	
Anticoagulant use				
Apixaban	64 (39.26%)	53 (42.74%)	11 (28.21%)	0.089 ^¶^
Edoxaban	34 (20.86%)	27 (21.77%)	7 (17.95%)	
Rivaroxaban	60 (36.81%)	42 (33.87%)	18 (46.15%)	
Warfarin	5 (3.07%)	2 (1.61%)	3 (7.69%)	
Antiarrhythmic use				
Amiodarone	100 (61.35%)	71 (57.26%)	29 (74.36%)	0.145 ^¶^
Propafenone	60 (36.81%)	50 (40.32%)	10 (25.64%)	
Sotalol	3 (1.84%)	3 (2.42%)	0 (0.00%)	
CRP	3 (2–8)	3 (2–5.55)	4.1 (2.2–14.7)	0.017 ^‡^
Albumin	42.49 ± 3.69	42.57 ± 3.71	42.23 ± 3.68	0.620 ^†^
Creatinine	0.93 (0.80–1.05)	0.95 (0.80–1.06)	0.89 (0.73–1.01)	0.258 ^‡^
Uric acid	5.74 ± 1.44	5.55 ± 1.32	6.35 ± 1.63	0.002 ^†^
Triglyceride	160 (121–200)	163.5 (118–198)	152 (128–215)	0.396 ^‡^
Total cholesterol	195 (165–217)	197 (168–217)	191 (133–214)	0.176 ^‡^
HDL-C	42.95 ± 9.79	43.30 ± 9.76	41.87 ± 9.93	0.430 ^†^
LDL-C	115.19 ± 33.44	117.32 ± 31.44	108.44 ± 38.81	0.149 ^†^
Hemoglobin	13.27 ± 1.83	13.31 ± 1.87	13.14 ± 1.69	0.613 ^†^
Platelet (×10^3^)	238.74 ± 76.54	238.69 ± 77.39	238.87 ± 74.74	0.990 ^†^
Neutrophil (×10^3^)	4.60 (3.85–6.20)	4.72 (3.79–6.42)	4.57 (3.89–5.72)	0.770 ^‡^
Lymphocyte (×10^3^)	2.17 ± 0.87	2.17 ± 0.92	2.15 ± 0.68	0.901 ^†^
Monocyte (×10^3^)	0.66 ± 0.24	0.67 ± 0.24	0.65 ± 0.21	0.690 ^†^
Uric acid-to-HDL-C ratio (%)	14.09 ± 4.81	13.43 ± 4.22	16.19 ± 5.91	0.002 ^†^
Uric acid-to-creatinine ratio	6.34 ± 1.90	6.13 ± 1.93	7.03 ± 1.67	0.009 ^†^

Descriptive statistics were presented using mean ± standard deviation for normally distributed continuous variables, median (25th percentile–75th percentile) for non-normally distributed continuous variables, and frequency (percentage) for categorical variables. ^†^ Student’s *t* test, ^‡^ Mann–Whitney U test, ^#^ Chi-square test, ^§^ Fisher’s exact test, ^¶^ Fisher–Freeman Halton test, * Significantly different category for variables with multiple categories. Abbreviations; AF: Atrial fibrillation, CRP: C reactive protein, HDL-C: High-density lipoprotein cholesterol, LDL-C: Low-density lipoprotein cholesterol.

**Table 2:** Performance of uric acid-to-HDL-C ratio and uric acid-to-creatinine ratio to predict recurrence of atrial fibrillation, ROC curve analysis.

	Uric Acid-to-HDL-C Ratio (%)	Uric Acid-to-Creatinine Ratio
Cut-off	>15.1	>6.45
Sensitivity	61.54%	71.79%
Specificity	70.97%	61.29%
Accuracy	68.71%	63.80%
PPV	40.00%	36.84%
NPV	85.44%	87.36%
AUC (95% CI)	0.665 (0.562–0.768)	0.671 (0.572–0.770)
*p*	0.002	0.001

Abbreviations; AUC: Area under ROC curve, CI: Confidence interval, NPV: Negative predictive value, PPV: Positive predictive value, ROC: Receiver operating characteristic.

**Table 3:** Summary of patients’ characteristics, treatment protocols, and laboratory measurements with regard to uric acid-to-HDL-C ratio.

	Uric Acid-to-HDL-C Ratio (%)		
	Low, ≤13.57 (n = 82)	High, >13.57 (n = 81)	*p*
Age, years	56.67 ± 12.74	58.32 ± 9.44	0.349 ^†^
Sex			
Female	38 (46.34%)	28 (34.57%)	0.126 ^#^
Male	44 (53.66%)	53 (65.43%)	
Weight, kg	81.74 ± 13.57	87.69 ± 13.38	0.005 ^†^
Height, cm	170.16 ± 9.96	170.32 ± 9.53	0.915 ^†^
Body mass index, kg/m^2^	28.19 ± 3.74	30.28 ± 4.37	0.001 ^†^
Smoking status			
Non-smoker	44 (53.66%)	44 (54.32%)	0.323 ^#^
Ex-smoker	25 (30.49%)	18 (22.22%)	
Active smoker	13 (15.85%)	19 (23.46%)	
Diabetes mellitus	17 (20.73%)	21 (25.93%)	0.549 ^#^
Hypertension	38 (46.34%)	49 (60.49%)	0.070 ^#^
Cerebrovascular disease	5 (6.10%)	6 (7.41%)	0.983 ^#^
Heart failure	8 (9.76%)	4 (4.94%)	0.380 ^#^
Coronary artery disease	18 (21.95%)	18 (22.22%)	1.000 ^#^
CHA_2_DS_2_-VASc score	2 (0–3)	2 (1–3)	0.912 ^‡^
Ejection fraction	60 (55–62)	60 (55–62)	0.655 ^‡^
Left atrium diameter	42.54 ± 5.31	42.89 ± 2.84	0.598 ^†^
AF type			
Paroxysmal	39 (47.56%)	36 (44.44%)	0.252 ^#^
Persistent	31 (37.80%)	25 (30.86%)	
Long persistent	12 (14.63%)	20 (24.69%)	
Intervention			
Radiofrequency ablation	36 (43.90%)	35 (43.21%)	0.929 ^#^
Cryoablation	46 (56.10%)	46 (56.79%)	
Anticoagulant use			
Apixaban	36 (43.90%)	28 (34.57%)	0.151 ^¶^
Edoxaban	20 (24.39%)	14 (17.28%)	
Rivaroxaban	25 (30.49%)	35 (43.21%)	
Warfarin	1 (1.22%)	4 (4.94%)	
Antiarrhythmic use			
Amiodarone	47 (57.32%)	53 (65.43%)	0.438 ^¶^
Propafenone	34 (41.46%)	26 (32.10%)	
Sotalol	1 (1.22%)	2 (2.47%)	
CRP	2.95 (2–8.2)	3.2 (2.1–6.8)	0.266 ^‡^
Albumin	42.42 ± 3.62	42.55 ± 3.79	0.826 ^†^
Creatinine	0.89 (0.70–1.03)	0.94 (0.85–1.10)	0.007 ^‡^
Uric acid	4.84 ± 1.08	6.66 ± 1.15	<0.001 ^†^
Triglyceride	144 (112–190)	180 (137–214)	0.004 ^‡^
Total cholesterol	199.5 (180–216)	190 (147–217)	0.097 ^‡^
HDL-C	47.60 ± 10.61	38.25 ± 5.98	<0.001 ^†^
LDL-C	118.94 ± 27.59	111.40 ± 38.27	0.151 ^†^
Hemoglobin	13.02 ± 1.81	13.53 ± 1.82	0.070 ^†^
Platelet (×10^3^)	246.77 ± 76.21	230.60 ± 76.48	0.178 ^†^
Neutrophil (×10^3^)	4.36 (3.68–5.94)	5.00 (4.07–6.42)	0.085 ^‡^
Lymphocyte (×10^3^)	2.06 ± 0.71	2.28 ± 0.99	0.109 ^†^
Monocyte (×10^3^)	0.64 ± 0.23	0.68 ± 0.24	0.289 ^†^
Uric acid-to-HDL-C ratio (%)	10.53 ± 2.56	17.69 ± 3.74	<0.001 ^†^
Uric acid-to-creatinine ratio	5.80 ± 2.07	6.90 ± 1.55	<0.001 ^†^
Recurrence	12 (14.63%)	27 (33.33%)	0.009 ^#^

Descriptive statistics were presented using mean ± standard deviation for normally distributed continuous variables, median (25th percentile–75th percentile) for non-normally distributed continuous variables, and frequency (percentage) for categorical variables. ^†^ Student’s *t* test, ^‡^ Mann–Whitney U test, ^#^ Chi-square test, ^¶^ Fisher–Freeman Halton test. Abbreviations; AF: Atrial fibrillation, CRP: C reactive protein, HDL-C: High-density lipoprotein cholesterol, LDL-C: Low-density lipoprotein cholesterol.

**Table 4:** Significant factors independently associated with recurrence and multivariable logistic regression analysis.

	Model 1		Model 2		Model 3	
	OR (95% CI)	*p*	OR (95% CI)	*p*	OR (95% CI)	*p*
Left atrium diameter	1.227 (1.074–1.402)	0.003	1.212 (1.063–1.381)	0.004	1.189 (1.039–1.360)	0.012
AF, Long persistent	2.633 (1.061–6.533)	0.037	2.660 (1.050–6.740)	0.039	2.722 (1.065–6.959)	0.037
CRP	1.013 (0.997–1.029)	0.104	1.013 (0.998–1.028)	0.081	1.018 (1.003–1.032)	0.015
Uric acid	1.464 (1.095–1.958)	0.010				
UHR (%), >15.1			3.824 (1.682–8.693)	0.001		
UCR, >6.45					4.340 (1.768–10.652)	0.001
Nagelkerke R^2^	0.265		0.290		0.296	

Abbreviations; AF: Atrial fibrillation, CI: Confidence interval, CRP: C reactive protein, HDL-C: High-density lipoprotein cholesterol, OR: Odds ratio, UCR: Uric acid-to-creatinine ratio, UHR: Uric acid-to-HDL-C ratio.

## Data Availability

The data supporting this study are available from the corresponding author upon reasonable request.
